# To tell or not to tell … the patient about potential harm

**DOI:** 10.3389/fnume.2023.1258960

**Published:** 2023-09-05

**Authors:** Timothy L. Bartholow

**Affiliations:** Independent Practitioner, Madison, WI, United States

**Keywords:** extravasations, infiltrations, healthcare transparency, patients, clinical obligations, reporting

## Abstract

Extravasation, as distinct from infiltration, is when a potentially toxic agent (e.g., radiographic contrast, chemotherapy, anesthesia or radionuclide) is unintentionally administered to the surrounding tissue instead of directly into the vein. There is an expectation for vascular access in interventional medicine across nearly all specialties that this high frequency, study/treatment critical procedure needs to occur with rare failure and that this failure rate should be characterized in quality assurance. This opinion piece, written by a family practitioner who has served as the chief medical officer for a not-for-profit payer, reflects on our responsibility to be aware as clinicians of known potential harm and disclose to patients before a risk has occurred and if harm has occurred. In this paper, clinical obligations of reporting will be reviewed, which are necessary to maintain and enhance our trust with our patients. In the second half, the perspectives of a not-for-profit payer chief medical officer will be considered.

## Introduction

*Our prime purpose in this life is to help others.*

*And if you can't help them, at least don't hurt them.*

*Dalai Lama*

The last several decades have seen a new and historic ability to identify abnormal vascular and tissue patterns with a variety of infusions, including radionuclides, which have advanced important diagnostic and treatment opportunities. On occasion, infusion does not go as planned. Extravasation occurs when potentially dangerous fluids are not infused into the intended intravenous environment, either because the intended intravascular cannula wholly missed the vessel, went through it, or once properly placed has been removed from the vessel by inadvertent movement. Adjunctive maneuvers during placement, like blood flash-back, easy flushing, ultrasound, and others, individually or in combination are simple, generally inexpensive and well-established diligent vascular access techniques to confirm proper placement. There has been wide variation of rates reported for extravasation, with a variety of harm ranging from the patient being unaware to tissue radionecrosis (1, 2). We applaud the work of members of the American College of Radiology who have established the National Radiology Data Registry to document and optimize successful vascular access for contrast studies using computed tomography (3–5). I suggest that patients will similarly benefit if extravasation of radionuclide is subjected to a similar intent to identify, remediate the individual event, and to implement process changes including education to reduce the rate of radionuclide infusion.

## Discussion

In this opinion piece, I will discuss the surprising debate about extravasation of radionuclide, whether to detect or not, whether to tell the patient before or after, and if to bill third party payers. In the past 3 decades, as quality assurance has grown in systematic capability in Medicine and as the patient safety movement has tangibly improved the care environment, it is predictable that early adopter specialties/disciplines would lead late adopters. Extravasation as a medical misadventure has now been mitigated by processes and education in most of medicine and and now extravasation reduction needs to be a more uniform expectation.

### Extravasation and the patient physician relationship

First, let me recall a personal experience from my work at the “wet bench.”

*In St. Louis, reagents were often expensive, some were carcinogenic or otherwise dangerous. Each technician who was going to be handling radioactive reagents completed a standard training during which “There is no safe level of radiation” was emphasized. Periodically across 30 labs on the same floor, there would be a radioactive reagent droplet contamination and we would follow rigorous decontamination process, including Nuclear Regulatory Commission defined documentation, including assessments before and after decontamination complete with Geiger counter readings. All radioactive waste was disposed to red plastic radioactive hazardous material containers, that would be uniquely handled and removed to the special trash truck for disposal to a special and expensive site. In the lab, full compliance was expected under the risk of employment separation and civil penalties for the organization.*

In clinical environments, medical physicists use complex dosimetry to calculate the exact dose of infused radionuclide to known digits of significance for the route of administration (6, 7). The physicist seeks to provide an effective diagnostic or generally larger treatment dose of whole body distributed radionuclide, specific to a successful infusion through a specific site, while also minimizing harm. This precisely calculated radionuclide is transported from production to the bedside in a lead pig (a lead container used to shield against radiation), to protect the technician and other staff. If this agent is spilled, rigorous procedures are required to decontaminate and to document remediation. At the patient bedside, we generally place an intravenous catheter. Most times, but not always, the radionuclide is successfully administered. However, we do not routinely effectively monitor to see if an extravasation has occurred that would defeat the exacting efforts of the nuclear medicine team to deliver the intended dose as prescribed. The careful dose calculation, the careful physical movement of radionuclide are juxtaposed to the paradoxical non-assessment of whether the infusion was or was not successful.

*An all-too-common circumstance: A senior patient presents in presumed cancer remission. The patient has non-descript back pain. A technetium bone scan is negative. After months of persisting pain, a repeat bone scan is completed and ultimately identifies a lesion at the site of pain.*

In these circumstances which have happened too frequently in my career, was the initial bone scan technically flawless and the study authoritatively identified the absence of metastatic disease? Or had the initial study suffered from unidentified extravasation reducing the potential signal of a metastatic lytic lesion? These patients often don't report more than irritating discomfort at the injection site. Physicians will recognize among technicians that some technicians will have greater or lesser confidence in their individual ability to place an intravenous cannula. If we study the infusion site, we can know immediately if the radionuclide is distributed. Without this injection site surveillance, the treating physician does not know, the radiologist does not know, and the patient does not know, if extravasation did or did not happen. Even without knowing this, a bill is virtually always sent to the insurer.

Reflecting on this case and several others I wonder how many studies with unrecognized extravasation miss a subtle emerging finding (8)? What is our responsibility to look for and characterize the extravasation and report it to patients and the clinical team when it has happened to know how it may alter diagnosis and treatment? If we identified extravasation, would we enroll these patients in a patient safety organization registry to be followed for the long term? Would we repeat the study to assure the best opportunity of diagnosis or treatment? If an extravasation occurs, should the patient and the insurer be charged?

#### What are the clinical obligations to patients?

Fiduciary (Trust) responsibility of the patient—physician relationship (9):

*The Fiduciary Principle*

*The doctor–patient relationship is spoken of sacredly as a fiduciary relationship, a relationship based on trust. That trust must be earned, at the least by the hard work required to acquire the skills necessary to apply the art. But it must also be earned by the consistent application of attentive response to the patient's needs, what we call “responsibility”. Unlike law or business, where the trustee may act for the client, in medicine the physician must act with the patient and with the patient’s consent, implying a partnership. That action may be paternalistic, but if that concern verges on control, the modern patient may well lose trust and confidence.*

Once the clinician believes that an intervention like radionuclide study is of greater benefit than burden, when is it desirable to inform a patient of the likely benefits and any potential risk so that the patient can provide authentic informed consent (10)? For the patient to be fully informed, the physician would share the likelihood of incomplete intervention, and the likelihood of harm, and describe what we do to avoid both. This potential harm would be assessed by each individual patient in the context of their belief systems and lived experience. Is our duty to informed consent satisfied when this known detail is not shared with patients, presuming they wish to know? How do we make a diligent opinion about a finding or treatment response if we do not know if extravasation occurred? If we know that an extravasation has occurred, we can mitigate the exposure, inform the patient, enroll them in a registry and we can assess the technician's training, remediating where indicated. If we do not systematically surveil for this success factor, how can we systematically reduce the risk of extravasation? Does the patient have a right to know these vulnerabilities if the medical service chooses not to surveil for extravasation?

#### When harm is potential but is not assessed, when underdiagnosis is possible but is not disclosed, is physician fiduciary obligation (patient trust) met?

Extravasation occurs between 1% and just less than 30% of the time (11). The Nuclear Regulatory Commission believes that there are about 11,000 infusions daily (261 weekdays annually) with “extensive extravasation”, 1% resulting in something less than 30,000 cases of extravasation per year (5). Many of these may result in surpassing the 0.5 Sievert threshold required for reporting, given “worst case” estimations of local tissue exposure when extravasation occurs (12). Given that, shouldn't each patient undergoing a radionuclide study be told about the benefits of a successful radionuclide infusion, along with the known local rate of ineffective vascular access, with or without harmful outcomes, if informed consent is authentically the goal?

#### When harm is known, is there an obligation to report to the patient, to avoid withholding information or mis-informing?^1^

*The doctor-patient relationship is fiduciary in nature, meaning that it is based on the patient's trust or confidence in the doctor. Once established, this relationship creates certain obligations or duties that the doctor owes the patient. One of the basic duties of physicians is to tell patients the truth about their diseases or conditions. Exceptions are allowed in certain circumstances if knowing the truth might be medically harmful to the patient. There are no exceptions, however, to the obligation to reveal the nature of adverse outcomes. Patients are absolutely entitled to a frank disclosure of the facts concerning their cases, especially when the results are adverse. Failure to provide a forthright account of the events, either by withholding information or by providing misleading information, is known as fraudulent concealment. This creates new and serious complications for the physician that are separate and distinct from the initial complication.*

Physician non-disclosure to the patient of adverse reactions or outcomes, including not disclosing that the care team could know but did not assess for known adverse outcomes, may not earn the trust of, nor fulfill the fiduciary duty to, patients (13). When non-disclosure is evident to the patient, it may feel like betrayal, leaving the patient unable to invest trust in the current team and by projection to other current or future medical professionals. When non-disclosure happens, in the best of circumstances, the next hard recommendation from the physician to the patient will be clouded by the uncomfortable uncertainty of “Would they tell me if they knew?” The AMA speaks directly to this circumstance in the Code of Ethics, 2.1.3^2^:

*Truthful and open communication between physician and patient is essential for trust in the relationship and for respect for autonomy. Withholding pertinent medical information from patients in the belief that disclosure is medically contraindicated creates a conflict between the physician's obligations to promote patient welfare and to respect patient autonomy.*

In addition to duty to each individual patient, active assessment for extravasation and registration where extravasation has occurred could lead to systematic process improvements resulting in extravasation reductions like have been seen in the laudable efforts around Contrast CT (14).

The Patient Safety movement in the United States, including at the University of Michigan, Institute for Healthcare Improvement Lucian Leape Institute, and others, has recommended a clinical culture of non-punitive reporting (15). In these environments, safety issues are ideally reviewed by Patient Safety Organizations which are explicitly designed to disclose details of unsuccessful care to the patient and to review internally without legal discovery, for the purpose of optimal process correction. These protections have been patterned after improvement efforts in industry where non-punitive reporting results in faster improvement in safety and quality.

Finally, the question of radionuclide extravasation needs to be solved in a framework of national policy decision making that involves physicians, nuclear physicists, and staff who administer the reagents. Some will rightly say that radionuclide extravasations, especially diagnostic, are low volume exposures that happen for only a minority of patients, resulting in uncommonly observed harm, and whose monitoring would require incrementally costly administrative steps. When we choose not to assess for extravasation when there is a known, if low, procedure failure rate and consequent diagnostic/treatment imprecision if not harm, and we choose as a matter of policy to not fully inform the patient, when are these same rationales to be used in other areas of clinical service with our neighbors, our families, ourselves? It is not just the choices we are making with not monitoring and reporting radionuclide extravasation; it is that physician and other clinician medical decision making should vigilantly defend the patient's full understanding of what worked and what did not and that we sacredly do what is best for them, including diligently reducing harm.

### Extravasation and the provider payer relationship

What is the perspective on extravasation of a payer chief medical officer?

Too often, an understandable if unproductive cynicism exists between provider system care teams and those that make payment. Earnest, diligent work is required of both provider and payer to protect patients from medical misadventure and indeed the toxicity of unnecessary cost. When either provider or payer underperforms this responsibility, people are harmed.

From the perspective of at least this chief medical officer who previously co-owned a clinic with partners for over 16 years, I appreciate the effort and complexity of delivering safe care. This takes the form of non-punitive reporting of opportunities, the redesign of the care environment with safety tools (checklists, pause before surgery), quality committees and other activities. Those who steward this safe, reliable value stream deserve and require sufficient resources in current environments where there is competition for resources, financial constraints, and revenue-cycle focus.

The purchaser of any product expects that the product will do what is described and clearly will not harm the consumer. In the complexity of health care, there are occasionally unintended outcomes, at times without harm and at other times, with harm. In order to fulfil the fiduciary obligation and earn and defend patient trust, if unintended outcomes occur, the product/service provider needs to notify the patient and mitigate any harm where possible. Sometimes this is as simple as acknowledging a mistake and describing action to have it not repeatedly happen to others in the future. Sometimes, this is litigated to test if the care received was the standard of care for a community. Several important efforts have been undertaken nationally around *I'm Sorry* legislation^3^ which has reduced litigation, while appearing to allow systems to more quickly engage and more fully focus on harm reduction. Let's also not forget that when the patient is harmed, the care team is very often harmed too, knowing that they did not achieve their best for the patient they care for. Worsened by the challenges of COVID-19, a sense of failure resulting from suboptimal outcome if unaddressed can lead to a more pervasive sense of clinician powerlessness and despair further contributing to poor outcome, burnout or worse.

The public knows that error will happen even with the greatest diligence, but avoiding learning from that error is not likely to be forgiven by patients, juries, administrative judges, or serious CMOs. If the patient who is harmed is not the ultimate payer for the service, the purchaser paying for the product is focused on the financial remedy for harm, express or implied warrantee, and demonstration that the provider is monitoring to achieve patient safety. Inclusion in a network infers by ostensible agency that the payer believes the provider practices care that is necessary, appropriate, safe and reliable for plan members. If this is not true, or the provider is not engaging in continuous improvement, or is avoiding full informed consent, or declines to disclose harm to the patient, the payer is responsible to review, to initiate a conversation with providers about the harm, and if necessary to exclude from a network. Intravenous vascular access occurs thousands of times per hour in America, and rigorous quality assurance programs are common for these maneuvers. Even in skilled hands with special tools, extravasation will occur in this very high volume procedure with a low but regular rate. Where nuclear medicine is often dependent on vascular access, applying programs to train to avoid extravasation and programs to monitor for its occurrence only seems prudent.

Payers pay every day for care provided in desperate circumstances with sometimes poor outcomes, acknowledging there is shared risk in the attempt of an urgent complex care event with sometimes catastrophic risks. However, in discrete controlled elective care environments, error is expected to be dramatically low, and when unsatisfactory delivery has occurred, payment for a technically incomplete procedure should not be requested, nor should it be paid. When extravasation rates are low, non-payment would only happen commensurate with the rate of extravasation. Said another way, non-payment would only be significant where extravasation rates were high. Such a policy would encourage clinical mastery, encourage quality improvement, and earn patient/employer/payer confidence, as well as clinician confidence.

When a procedure is susceptible to error, assessment for absence of error seems prudent. If error is known, confidence is earned if this poor outcome is disclosed to the patient and to the payer, and if the care team is encouraged to engage process improvement.

Beyond the legal concerns to payers, the annual economic impact in the United States of 30,000 extensive extravasations with incompletely documented toxicities, and uncertain loss of diagnostic insight, and therapeutic undertreatment at even $20,000 of cost per case is billions of dollars over 10 years (used in federal budgeting) and a self-inflicted loss of patient confidence (5). In studies undertaken in all-payer claims data bases, a reasonable estimate of the economic costs might include:

(1) Costs of repeating the procedure/treatment
(2) Costs of mitigating harm, immediate and delayed diagnosis of damage to local tissue
(3) Costs of delayed diagnosis, assuming that one event in 20–50 leads to catastrophic later stage treatment.

## Conclusions

Clinical, ethical, legal and financial imperatives invite American medicine to make ubiquitous vascular access optimally reliable and safe. Where it is not looked for, it appears that extravasation in nuclear medicine can result in potential nosocomial harm, incomplete documentation of adverse events, incomplete information to patients, uneven skill for health care system vascular access services, uncertain delay in diagnosis or treatment for patients, and harm to the care team when clinicians witness avoidable harm that is not avoided. We urge that all care environments continue vigilance around vascular access and that The Joint Commission and the Nuclear Regulatory Commission continue to challenge us to perform to our best potential. If spills of radionuclides in the wet lab environment are worthy of significant decontamination effort and documentation monitoring for extravasation (a “spill” into tissue) and maintenance of a patient registry seems advised. Any costs of repeated studies, harm, or delayed diagnosis or treatment is eclipsed by the loss of confidence and trust that will predictably result if we do not act in the best interest of the patient.

Finally, a personal note. Thank you to clinical care teams who help our families. If such a risk as radionuclide extravasation exists in the care of me or my family, you may surface concerns I hadn't thought of, and I may momentarily hesitate to proceed, but I will be comforted to know that your professional team has committed to having me and my family understand and authentically choose what we wish to do. And when I have another clinically difficult circumstance, I will be able to return to your advice knowing that you will be forthright with me.

In advance, thank you for being patient to hear my concern.

## Data Availability

The original contributions presented in the study are included in the article/Supplementary Material, further inquiries can be directed to the corresponding author.

## Footnotes

^1^ https://www.sciencedirect.com/science/article/pii/B9781416022152502465

^2^ https://code-medical-ethics.ama-assn.org/sites/default/files/2022-08/2.1.3.pdf

^3^ https://www.theclm.org/Magazine/articles/apology-laws-medical-malpractice/2172
